# Dopamine and Acetylcholine, a Circuit Point of View in Parkinson’s Disease

**DOI:** 10.3389/fncir.2017.00110

**Published:** 2017-12-22

**Authors:** Giorgio Rizzi, Kelly R. Tan

**Affiliations:** Biozentrum, University of Basel, Basel, Switzerland

**Keywords:** dopamine, acetylcholine, sub-circuits, Parkinson’s disease, optogenetics

## Abstract

Data from the World Health Organization (National Institute on Aging, 2011) and the National Institutes of Health (He et al., 2016) predicts that while today the worldwide population over 65 years of age is estimated around 8.5%, this number will reach an astounding 17% by 2050. In this framework, solving current neurodegenerative diseases primarily associated with aging becomes more pressing than ever. In 2017, we celebrate a grim 200th anniversary since the very first description of Parkinson’s disease (PD) and its related symptomatology. Two centuries after this debilitating disease was first identified, finding a cure remains a hopeful goal rather than an attainable objective on the horizon. Tireless work has provided insight into the characterization and progression of the disease down to a molecular level. We now know that the main motor deficits associated with PD arise from the almost total loss of dopaminergic cells in the substantia nigra pars compacta. A concomitant loss of cholinergic cells entails a cognitive decline in these patients, and current therapies are only partially effective, often inducing side-effects after a prolonged treatment. This review covers some of the recent developments in the field of Basal Ganglia (BG) function in physiology and pathology, with a particular focus on the two main neuromodulatory systems known to be severely affected in PD, highlighting some of the remaining open question from three main stand points:

- Heterogeneity of midbrain dopamine neurons.

- Pairing of dopamine (DA) sub-circuits.

- Dopamine-Acetylcholine (ACh) interaction.

A vast amount of knowledge has been accumulated over the years from experimental conditions, but very little of it is reflected or used at a translational or clinical level. An initiative to implement the knowledge that is emerging from circuit-based approaches to tackle neurodegenerative disorders like PD will certainly be tremendously beneficial.

## Introduction

**Figure 1:**
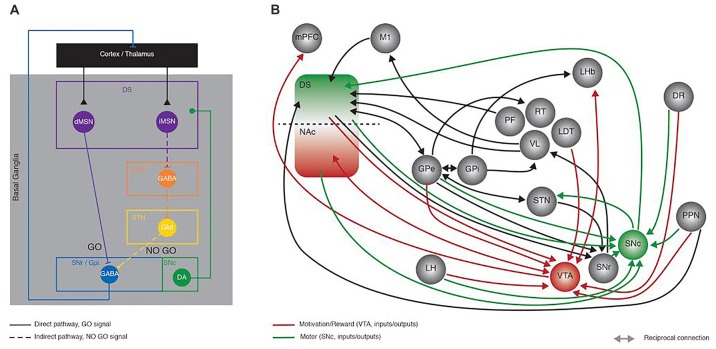
Recent developments in Basal Ganglia (BG) circuit organization. This figure aims at highlighting the increased knowledge collected in the last years in the network organization of the BG with the Delong model proposed in the late 90’s as a starting point. **(A)** Schematic representing the Delong rate model of BG organization where activation of the direct (full line) and indirect (dotted line) pathways has opposing effects onto the thalamo-cortical loop. In the direct pathway, dMSNs inhibit the output structures (SNr/GPi), sending a GO motor signal; conversely the iMSNs disinhibit the subthalamic nucleus (STN) via the inhibition of the GPe, ultimately providing a NO GO motor signal. **(B)** Simplified representation of the connectivity between BG nuclei, showing some of the recently described synaptic contacts (reported in the current review as examples of the advances made in the field), as revealed by monosynaptic rabies anatomical mapping and/or optogenetic and electrophysiological dissection. Green and red connection lines represent the direct inputs and outputs to SNc and VTA, highlighting the parallel between motor and motivation/reward related circuits, respectively. DS, Dorsal striatum; NAc, nucleus accumbens; dMSN, direct pathway medium size spiny neurons; iMSN, indirect pathway medium size spiny neurons; GPe, Globus Pallidus external; GPi, Globus Pallidus internal segment; STN, subthalamic nucleus; SNr, Substantia Nigra pars Reticulata; SNc Substantia Nigra pars Compacta; mPFC, medial prefrontal cortex; M1, primary motor cortex; LH, lateral hypothalamus; PF, parafascicular thalamus; RT, reticular thalamus; VL, ventro-lateral thalamus; LDT, latero-dorsal thalamus; LHb, lateral habenula; DR, dorsal raphe; PPN, Pedunculopontine Nucleus; VTA, ventral tegmental area.

The Basal Ganglia (BG) are a group of nuclei comprising the striatum, the Globus pallidus (internal—GPi and external—GPe segments), the subthalamic nucleus (STN) and the substantia nigra (pars reticulata—SNr and pars compacta—SNc; Figure 1A). Together, these subcortical areas process sensorimotor information to allow proper movement generation, including action selection, planning, execution and orientation of locomotion (Albin et al., 1989). Beyond its well acknowledged role in motor function, recent studies have demonstrated the BG implication in motivated behavior (Kravitz and Kreitzer, 2012; Kravitz et al., 2012) which has been for a long time, attributed exclusively to the reward system. The importance of adequate BG function is highlighted in clinical evidence from BG related pathologies such as Parkinson’s disease (PD), where SNc dopamine (DA) neurons degenerate (Hornykiewicz, 1975a,b), leading to a multitude of incapacitating symptoms ranging from motor dysfunctions to cognitive disabilities (Lang and Lozano, 1998).

The main theory describing the anatomical organization of BG nuclei or the so-called rate model (DeLong, 1990) is based on a series of clinical observations diagnosing specific brain lesions, experimental and post mortem human anatomy as well as neurochemical analyses. This model describes two parallel circuits, the direct and indirect pathways, both being modulated by SNc DA. Activation of the direct pathway is motor-permissive (GO), conversely, activation of the indirect pathway has motor-suppressive (NO GO) effects (Figure 1A). This anatomical model is supported at the molecular level by the well described DA neuromodulation and signaling cascades involved, particularly the opposing effects of DA onto direct and indirect medium size spiny neurons (MSNs; dMSNs, iMSNs; respectively). For an in-depth description of the BG anatomy we suggest the following reviews (Alexander et al., 1986; Albin et al., 1989; DeLong, 1990).

### Latest Anatomical Observations

The emergence of novel tools like circuit-based optogenetic manipulations, *in vivo* single cell calcium imaging and monosynaptic retrograde mapping (Boyden et al., 2005; Watabe-Uchida et al., 2012; Cui et al., 2013) that allow the probing of behavioral functions with a much higher degree of specificity (Emiliani et al., 2015; Whissell et al., 2016), has yielded in the past decade, a number of observations that on one hand confirmed the principle of the GO vs. NO-GO hypothesis (Kravitz et al., 2010) but also further depict the complexity of the BG function and organization, ultimately augmenting the original rate model. Without going into the details of all the recent advances in the BG field, here we provide a few representative examples (Figure 1B).

A very recent single cell transcriptional study has revealed the existence of unreported pathways, namely entopenduncular parvalbumin (EP-PV) cells projecting to the lateral habenula (LHb) and the motor thalamus whereas EP-SOM (somatostatin) cells projecting exclusively to the LHb (Wallace et al., 2017).

Several routes of cortical information flow via indirect pathways have now been identified. In addition to the inhibition of the STN (Mallet et al., 2012), the GPe also has inhibitory control over the GPi (Smith et al., 1998), the reticular thalamic nucleus (RT; Smith et al., 1998) and directly the SNr (Saunders et al., 2016).

There is evidence of a high degree of reciprocal connectivity between indirect pathway BG nuclei (GPe, STN, GPi, SN; Smith et al., 1998; Mallet et al., 2012; Saunders et al., 2016) some of which provide direct feedback outputs to cortical regions (Saunders et al., 2015). The identification of synaptic connectivity between two brain nuclei however, does not necessarily lead to trivial consequences at the functional level. This is very well exemplified by the lack of synchronous firing of the GPe and the STN despite their robust connectivity (Baufreton et al., 2009). These novel connections should therefore be also functionally probed.

In relation with the activity of direct and indirect pathways, several studies have identified the presence of lateral inhibitory projections, particularly showing how there is not only D1-D1 and D2-D2 inhibition, but also reciprocal inhibition with the indirect onto direct (D2-D1) being much stronger (Taverna et al., 2008). These reciprocal connections are also differentially modulated by DA itself (Tecuapetla et al., 2009). An interesting yet undetermined aspect regarding these observations is the importance of these mutual inhibitory connections for proper motor behavior. Whether a further level of DA modulation at these sites, going beyond the canonical striatal DA modulation is necessary and to what extent, and if the loss of such modulation would lead to motor impairments is still unexplored territory.

### Latest Behavioral Observations

In addition to pure anatomical progress, there has also been a correlative increase in the knowledge at the functional level. Only few studies combine the investigation of anatomical circuitry and their impact on behavior. Lately a novel pathway linking BG nuclei to non-BG nuclei has been described and reported to process outcome evaluation. Specifically, habenular projecting Globus Pallidus neurons integrate bidirectionally reward and punishment expectation-related information (Stephenson-Jones et al., 2016).

*In vivo* calcium imaging in awake behaving mice has tremendously helped refining our knowledge. Action selection relies on the fact that both direct and indirect pathways are co-active and collaborate, with one promoting an action while the other one suppresses competing actions (Fan et al., 2012; Cui et al., 2013; Freeze et al., 2013; Isomura et al., 2013; Jin et al., 2014; Tecuapetla et al., 2016).

Additional evidence regarding the behavioral function of the BG has emerged from the pathology domain. Some clinical observations support the rate model yet others highlight its limitations. For example, STN deep brain stimulation improves motor behavior in PD patients (Fasano et al., 2012), and GPi unilateral pallidotomy improves cardinal motor signs, including tremor, rigidity, bradykinesia (Vitek et al., 2003), reason why both nuclei are thought to be hyperactive in PD. Apomorphine (non-selective DA receptors agonist) however, decreases the firing rate of the GPi but not the STN (Bergman et al., 1990; Aziz et al., 1991; Lang et al., 1997; Levy et al., 2001), despite the presence of DA receptors in both regions (Sun et al., 2012; Galvan et al., 2014). Alternative and yet to be characterized mechanisms must therefore be involved, that allow a BG modulation of motor function at a higher-level bypassing direct molecular interactions.

We believe that a better knowledge of the circuitry and its dynamics in physiological and the direct comparison to pathological conditions will provide hints to understand the reported discrepancies but most of all help with the design of better targeted treatments. Although there are several debilitating BG-related diseases, here we will only take into account PD, considering that the basic principles discussed would also apply to other pathologies.

The cause of PD is still unknown but the loss of neuromodulation correlates with motor and cognitive symptoms. So far, extensive work has been dedicated to the investigation of different aspects of the disease such as the Lewy body lesions and alpha-synuclein aggregation (Schirinzi et al., 2016), as well as genetic factors (Tanner and Aston, 2000). We propose that yet another aspect could prove instrumental in designing effective therapies, and that is a circuit-based approach.

This review is not an exhaustive overview of literature, it rather aims at highlighting some of the remaining open questions about BG in physiology and pathology, with a focus on DA and Acetylcholine (ACh) neuromodulation, following three main stand points:

- Heterogeneity of midbrain DA neurons.
- Pairing of DA sub-circuits.
- Dopamine-ACh interaction.

### Molecular Signaling of Dopamine and Acetylcholine

DA and ACh are the two main neuromodulators fine tuning the activity of BG nuclei. The most striking argument in support of a critical role of DA and ACh in the BG comes from pathological conditions. At the diagnostic time point of PD, at least 70% of SNc DA neurons have degenerated, consequently, motor dysfunctions manifest (Hornykiewicz, 1975b). Concomitantly, and as demonstrated with post-mortem human brain histology, choline acetyltransferase (ChAT) expressing neurons also degenerate (Nakano and Hirano, 1984; Braak et al., 2004). Both the cell death and the reduction in cholinergic markers correlate strongly with a decline in cognitive abilities assessed through performance tests in PD patients (Perry et al., 1985). For this reason, current therapies target both the DA and ACh modulatory systems. Rivastigmine, a cholinesterase inhibitor has proven effective in ameliorating the cognitive dysfunction (van Laar et al., 2010).

**Figure 2:**
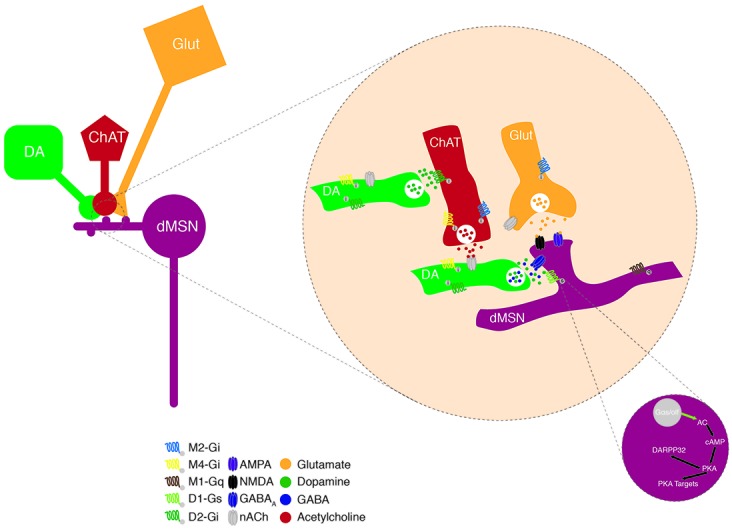
Neuromodulation of MSNs. Schematic representing the convergence of dopamine (DA) and ACh modulation on excitatory inputs onto a dMSN in the dorsal striatum (DS). DA terminals modulate excitatory neurotransmission forming synapses onto the neck of MSN spines; through two molecular mechanisms involving DA and the co-released GABA. These DAergic input is modulated by presynaptic nAChRs and this cholinergic control can itself be fine-tuned via the presence of presynaptic D2Rs. Activation of D1Rs, that are coupled to the Gαs protein, leads to the production of cyclic adenosine monophosphate (cAMP) via the adenylate cyclase (AC). This promotes the protein kinase A (PKA) function, which phosphorylates DARPP32, indirectly driving an increase in neuronal excitability via the activation of Ca^2+^ channels and NMDA receptors as well as the inhibition of K^+^ channels.

To understand how DA and ACh play such an important role in the BG physiology and dynamics, a first approach has yielded the molecular characterization of which receptors they activate and what intracellular signaling cascades are engaged to impact neuronal activity (Figure 2). DA receptors are G-protein-coupled receptors (GPCRs; Kebabian and Calne, 1979) that trigger two opposing signaling cascades depending on the G-protein they activate. The D1 and D5 receptors, members of the D1-like family of DA receptors are coupled to Gsα/Golf, which activates adenylyl cyclase (AC), increasing the intracellular concentration of the second messenger cyclic adenosine monophosphate (cAMP). Conversely D2, D3 and D4 receptors, part of the D2-like family, are coupled to Giα, which inhibits the AC and therefore blocks the production of cAMP (Surmeier et al., 2007). Elevated levels of cAMP facilitate the activation of protein kinase A (PKA) which in turn, among other targets, phosphorylates DARPP32 (Blank et al., 1997; commonly used as a biochemical marker to identify MSNs; Fienberg et al., 1998), ERK and CREB, permitting the transcription of immediate early genes (IEG) and also phosphorylates NMDA receptor subunits (Hallett et al., 2006) to allow Ca^2+^ influx and hence, increase neuronal excitability (Carter and Sabatini, 2004). Lower levels of cAMP eventually lead to reduced DARPP32 activation and IEG transcription and decreased neuronal firing rate due to reduced Na^+^ currents (Schiffmann et al., 1995) and an enhanced K^+^ conductance (Pacheco-Cano et al., 1996). Through this molecular signaling cascade, DA amplifies or reduces the strength of glutamatergic synaptic drive coming from the Cortex/Thalamus, favoring the activity of dMSNs and decreasing that of iMSNs (Nicola et al., 2000).

ACh on the other hand, binds ionotropic or ligand gated nicotinic (nACh) channels and metabotropic muscarinic GPCRs. Both types exhibit different isoforms with specific cellular and sub-cellular locations. The nACh receptors are pentamers of 3α and 2β (3 and 9 neuronal isoforms, respectively) subunits. The striatal receptors are composed of the α4, α6, α7 and β2, β3 subunits (Wada et al., 1989). Those channels are mainly expressed at presynaptic glutamatergic terminals and once activated increase the Ca^2+^ and Na^+^ influxes contributing to further depolarizing these terminals, enhancing the release of neurotransmitters and neuromodulators. The muscarinic receptors are classified into two groups the M1-like (M1, M3 and M5) coupled to Gq and the M2-like (M2 and M4) coupled to Gi/o. Post-synaptically, activation of the M1-like receptors triggers the phospholipase C to increase the levels of IP3 leading to the release of Ca^2+^ from internal stores, which in turn can activate the transcription of various IEGs, and eventually increase neuronal excitability (Narushima et al., 2007; Kreitzer, 2009). Similarly, to the D2-like receptors, activation of the M2-like receptors inhibits the AC signaling cascade ultimately dampening neuronal excitability (Calabresi et al., 1998).

The sub-cellular location of DA and ACh receptors is critical to ensure fine and adequate tuning of the synaptic transmission (Figure 2). For example, most MSNs are simultaneously contacted by a dopaminergic and a glutamatergic input, forming the so-called triad, where DA terminal fibers form symmetric synapses on the neck of the dendritic spines whereas glutamatergic afferents contact the head of the spine and form asymmetric synapses (Smith et al., 1994). This triad architecture is proposed to function as a hetero-synaptic coincidence detector by which DA selectively influences the convergence of glutamate at individual spines (Yao et al., 2008).

### Heterogeneous Properties of Midbrain Dopaminergic Cells

The DA neuronal population has revealed heterogeneous in many aspects. The most striking evidence likely comes once more from the clinical observation that motor manifestations are primarily linked to the selective loss of DA neurons in the SNc (Brichta and Greengard, 2014), whereas the very similar DA neurons in the VTA demonstrate a higher degree of resistance to degeneration (Dauer and Przedborski, 2003) and don’t carry motor specific impairments. An intriguing question is the extent of the difference between the SNc and the VTA DA cells, and the underlying neurobiological substrates. It has been proposed that the vulnerability to degeneration could lie in the genetic or molecular profile of each cell group. Although comparative analyses showed that only less than 3% of the gene expression pattern is different (Grimm et al., 2004), a number of studies reported potentially interesting differences. Functional analysis showed more transcripts of metabolism, mitochondrial machinery, lipid vesicle mediated transport and kinase/phosphatase signaling in the SNc compared to VTA DA neurons (Chung et al., 2005; Greene et al., 2010), whereas a higher number of transcripts for proteins involved in synaptic plasticity, cell survival and migration are found in the VTA as compared to the SNc (Grimm et al., 2004). In terms of markers implicated in cell firing, an example of discrepancy is the potassium channel GIRK2 protein and mRNA levels, being less abundant in the VTA than in the SNc (Chung et al., 2005; Eulitz et al., 2007; Fu et al., 2012). Conversely, the Calbindin (CB) protein and mRNA levels are less abundant in SNc than VTA (Chung et al., 2005; Greene et al., 2010). Furthermore, core molecules that define a DA phenotype such as the dopamine transporter (DAT) and vesicular monoamine transporter type-2 (VMAT2) show variability in their expression levels (Sanghera et al., 1994; González-Hernández et al., 2004). Although it is not clear which of the SNc or the VTA neurons express more of these two markers due to discrepancies from different studies (Liang et al., 2004; Reyes et al., 2013), it is acknowledged that the levels are different. A thorough and comprehensive characterization of the distribution of these markers would provide a great tool for the investigation of midbrain DA neurons.

Extensive work has shed light onto the differences in the genomic and proteomic expression patterns of SNc and VTA DA neurons (Brichta and Greengard, 2014; Veenvliet and Smidt, 2014; Anderegg et al., 2015), however only few studies have correlated them with specific electrophysiological properties. CB correlates with specific electrophysiological properties mediated by hyperpolarization-activated cyclic nucleotide-gated (HCN) channels (Neuhoff et al., 2002) and an inverse correlation was reported with the probability of vesicle exocytosis (Pan and Ryan, 2012).

It is still not known if these differences are responsible for any protective or harmful effects towards degeneration. As for any other differences reported so far, we still lack enough compelling evidence, for example, to understand whether the high CB expression in VTA DA cells has a causal role in their resilience to PD induced degeneration. In addition, no clear correlation has been made to validate such proteins as early detection markers for PD.

DA neuronal signaling has recently become even more complex with the demonstration that some VTA cells have the ability to co-release different neurotransmitters. It has been reported that a subset of these neurons express tyrosine hydroxylase (TH) and do not project to the ventral striatum (VS) but to the LHb (Stamatakis et al., 2013). Furthermore, some VTA DA neurons can co-release glutamate at the level of the VS. This has been confirmed both *in vitro* (Sulzer et al., 1998; Joyce and Rayport, 2000; Dal Bo et al., 2004; Stuber et al., 2010) and *in vivo* (Chuhma et al., 2004; Hnasko et al., 2010).

Considering that DA can be released unaccompanied or co-released with different neurotransmitters, the functional/behavioral implication of each neurotransmission modality is unknown. Similarly, GABA and glutamate neurons projecting to DA innervated regions have been identified in the VTA. The impact of the co-released GABA or glutamate vs. their pure counterparts is also unanswered. Unless otherwise proven developmental left-overs are a possibility and would be responsible for the co-released forms. Another hypothesis postulates that it provides the temporal specificity required for reward prediction and/or incentive salience (Lapish et al., 2006, 2007). Mice lacking vglut2 in DA neurons show deficits in psychostimulant-induced locomotion, providing some evidence that the ability of midbrain DA neurons to co-release different neurotransmitters is crucial in some specific cases at the behavioral level. Another hypothesis is that the presence of main neurotransmitters in DA cells aids in the vesicular packaging of DA itself, boosting the strength of DA modulation (Hnasko et al., 2010; Wallén-Mackenzie et al., 2010).

Molecular heterogeneity in the form of the specific channel expression can lead to significant functional differences. Adult SNc DA neurons rely on L-type voltage gated Ca^2+^ channels Ca*v*1.3 for pace making, whereas VTA DA neurons use voltage-dependent Na^+^ channels (Chan et al., 2007). Further studies have investigated differences within the VTA populations converging anatomical, electrophysiological, and molecular properties (Lammel et al., 2014; Anderegg et al., 2015). A subset of DA cells exhibits unconventional fast-firing properties and low DAT/TH and DAT/VMAT2 mRNA expression ratios, and selectively projects to prefrontal cortex. These cells lack the functional somatodendritic Girk2-coupled D2 autoreceptors. In contrast, conventional slow-firing DA neurons only project to the lateral shell of the nucleus accumbens (Nac), they express higher DAT/TH and DAT/VMAT2 mRNA expression ratios as well as D2 autoreceptors (Lammel et al., 2008).

The cells lacking the D2 autoreceptors were difficult to classify, they are considered as DA insensitive DA neurons (Margolis et al., 2006; Lammel et al., 2008). Even though the controversy is still not clearly resolved, a recent study using TH-GFP transgenic mice (eGFP under the control of the TH promoter) may provide some clarifications (Krashia et al., 2017). Combining electrophysiological recordings with DA pharmacological application, it was shown that TH-GFP positive cells exhibit different properties (cell body size, firing frequency, Ih current, sensitivity to DA, cocaine and Met-enkephalin). Such variability is correlated with the cell location within a medio-lateral axis. For example, and more specifically; the vast majority of SNc (98%) and VTA (92%) TH-GFP positive cells are inhibited by DA, with VTA medial cells exhibiting the lowest sensitivity. The latter population could identify with the D2-lacking mesocortical subtype reported in Lammel et al. (2008).

Another example of functional heterogeneity in VTA DA neurons highlighted two subpopulations that while exhibiting no hyperpolarizing currents (Ih), respond differentially to rewarding vs. aversive stimuli. Medial shell projecting neurons show an increased AMPA/NMDA ratio in response to cocaine, whereas medial prefrontal cortex (mPFC) projecting cells go through this adaptation but in response to an aversive stimulus (hindpaw formalin injection). As a counterpart out of the Ih positive cells, the lateral shell projecting neurons respond to both types of stimuli, whereas nigrostriatal cells are insensitive to both (Lammel et al., 2011).

From a circuit point of view, it was shown that the LDT-VTA-Nac sub-circuit drives place preference, in contrast, the Lhb-VTA-mPFC pathway produces place aversion highlighting once more that neurons within the same midbrain population can have strikingly different properties (Lammel et al., 2012).

The knowledge in DA heterogeneity has been much more investigated in the VTA than in the SNc. However similar concepts can be applied to the SNc DA neurons. Particularly, SNc DA cell functional heterogeneity has been correlated with differences in dendrite architecture and afferent connectivity (Brown et al., 2009; Henny et al., 2012). Furthermore, K^+^-ATP channels gate bursting activity selectively in medial SNc DA neurons projecting to the dorsomedial striatum (DMS), but not in lateral SNc DA neurons, which project to the dorsolateral striatum (DLS; Schiemann et al., 2012).

Anatomical studies have reported input diversity with GABAergic afferents arising from the striatum, the GP and the SNr (Fujiyama et al., 2002; Boyes and Bolam, 2003), and glutamatergic inputs from the STN and the pedunculopontine nucleus (PPN; Bolam et al., 1991; Chatha et al., 2000). There is also evidence that some SNc DA cells project to BG nuclei other than the striatum (Smith and Kieval, 2000; Prensa and Parent, 2001), for example the STN (Cragg et al., 2004). Such heterogeneity in input and output connectivity could imply that just as for the VTA, subsets of SNc DA cells are embedded in different sub-circuits with specific and different relevant roles for behavioral outcomes. Interestingly, during goal-directed actions the DMS is more strongly recruited as opposed to the DLS which is engaged to a lesser extend (Gremel and Costa, 2013). It is tempting to speculate that the same difference in coding properties could be observed at the level of the SNc having subsets of cells recruited during goal-directed vs. habitual actions that would project to DMS and DLS, respectively.

SNc DA cells have also been shown to co-release GABA resulting in the inhibition of dorsal striatal MSNs (Tritsch et al., 2012, 2016). Once more the functional relevance and/or necessity of such co-released GABA is unknown.

An additional level of complexity hinting towards diverse functional roles of DA release encoded at different time scales has been recently reported. Using complementary micro-dialysis and voltametric methods during an adaptive-based decision-making task, it was found that the motivational vigor of an action is encoded within the tonic DA release in the time scale of minutes, whereas acute DA released in the time scale of seconds assigns the value to a reward (Hamid et al., 2016).

Understanding the heterogeneity within the SNc DA neuronal population including their electrophysiogical properties, circuit connectivity and hence behavioral specialization becomes even more relevant and necessary when considering the PD pathology. Placing all these observations of heterogeneous properties in the pathological domain will provide crucial information to identify markers/properties that determine the faith of DA cells once the disease is ongoing, and would provide opportunities for interventions with a diagnostic or therapeutic goal.

### Pairing of DA Sub-circuits

DA neurons in the VTA are thought to play important roles in motivation and reward (Ikemoto, 2007) whereas SNc DA neurons are important for sensorimotor function, motor execution and habit formation (Haber, 2003). For an in depth review of such functions, we suggest the following studies Graybiel et al. (1994); Hikosaka et al. (1999); Yin and Knowlton (2006).

In brief, pharmacological studies have shown that the rewarding effects of many addictive drugs are mediated by DA neurons localized in the VTA and projecting to the VS and limbic cortices (i.e., the mesolimbic DA system; Koob, 1992; Robbins and Everitt, 1996). This has been confirmed by optogenetic studies showing that the excitation of VTA DA neurons induces conditioned place preference in mice (Tsai et al., 2009), and rodents are able to learn instrumental responding to excite VTA DA neurons (Adamantidis et al., 2011; Witten et al., 2011; Pascoli et al., 2015). The mesolimbic DA system also plays a role in negative affect. Conditioned place aversion can be induced by DA pharmacology (Shippenberg, 1991) and also with optogenetic inhibition of VTA DA neurons (Stamatakis and Stuber, 2012; Tan et al., 2012).

Once again, the value of SNc DA neurons is reflected by motor dysfunction in PD as reported by DeLong (1990) and the effectiveness of DA pharmacology as a treatment (Connolly and Lang, 2014). In addition to motor deficits, PD patients also suffer a cognitive decline (Wolters and Francot, 1998; Duncan et al., 2014). Congruently, SNc DA cells are considered to be key for focusing attention on significant and rewarding stimuli, a requirement for the acquisition of new learned behaviors (Schultz, 1986; Schultz et al., 1997; Wise, 2009). Recent optogenetic studies have provided strong evidence supporting this theory. Mice learn to self-stimulate SNc DA neurons (Rossi et al., 2013) and it can be as rewarding as VTA DA photo-activation (Ilango et al., 2014). Furthermore, photo-inhibition of both cell types induces a similar aversion (Ilango et al., 2014). This raises the question of the differential impact of SNc vs. VTA DA neurons in modulating behavior, which is further supported by studies reporting both populations acting as reward prediction or teaching signals (Bromberg-Martin et al., 2010; Schultz, 2017; Watabe-Uchida et al., 2017).

**Figure 3:**
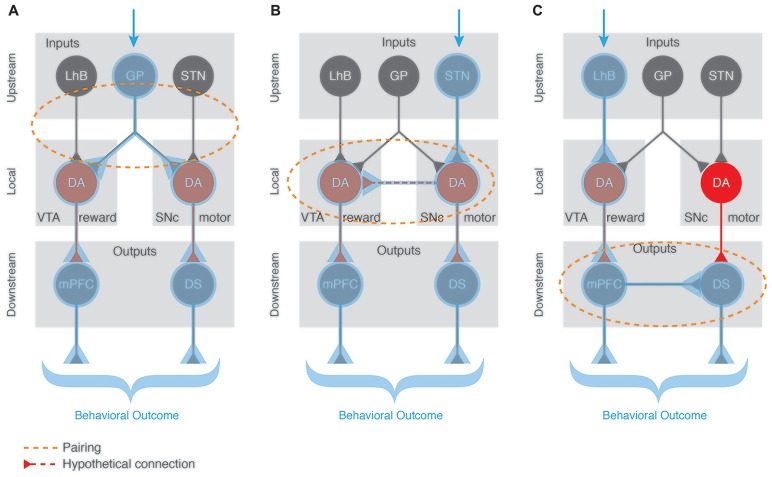
Pairing of functionally distinct dopaminergic sub-circuits. A salient external stimulus (blue arrow) can activate different pathways depending on the context and valence of the stimulus. We propose 3 different scenarios speculating on how the BG circuitry can be engaged to produce an appropriate and complex (reward and motor-based) behavioral response. **(A)** Upstream pairing scenario with a common input (e.g., GP) recruiting both VTA and SNc DA populations. When such a common input is activated, it synchronizes the DA release of VTA and SNc at their specific targets (mPFC and DS, respectively), hence recruiting these complementary pathways to engage both reward and motor programs for the execution of a task. **(B)** Local pairing via a so far unreported direct DA-DA interaction where the activation of one DA neuronal population induces the modulation of the other, hence engaging both complementary output pathways (mPFC and DS) to produce an elaborated behavioral outcome. **(C)** Downstream pairing scenario with mutual recruitment of output regions independently of the DA source. For example, the activation of VTA DA cells engages its specific output target (the mPFC) which itself activates the SNc DA neurons’ target (DS) independently of the release of DA from the SNc cells. Blue shading highlights the recruited regions for each scenario. Orange dotted shapes represent the location where the pairing occurs.

Any behavior is the sum of several components that are integrated or synchronized in order to allow the behavior to be generated adequately. This is also true for positive reinforcement where in the simplest of cases, a motor and a reward component play important roles. How is such integration happening? In other words, how does DA from the SNc and from the VTA collaborate to shape reinforcement? We hypothesize three scenarios:

1. DA must be released by both cell types at the appropriate targets within a very short time window to synchronize the activity of the circuits responsible for the behavioral sub-components. This would imply that the linking factor is located upstream of the VTA and the SNc DA cells (Figure 3A), and simultaneously recruits them. Anatomical mapping approaches have revealed common inputs such as the GP, DS, lateral hypothalamus (LH), dorsal raphe (DR) or PPN (Watabe-Uchida et al., 2012). Recent studies complement this approach by mapping monosynaptic inputs to subpopulations of DA neurons that project to specific brain areas (Beier et al., 2015; Lerner et al., 2015; Menegas et al., 2015). Even when DA-projection targets are specified, there is still monosynaptic inputs to SNc and VTA from numerous brain areas, some of which are overlapping (Ogawa et al., 2014). Such an overlap at the input level, favors the hypothesis that integration of VTA and SNc DA signal happens upstream via these common regions. We speculate that when these inputs are activated in response to external cues/stimuli, they synchronize the release of SNc and VTA DA at different targets, hence recruiting different yet specific pathways to engage both motor and reward programs for the execution of a reinforcement task.
2. Mutual local recruitment of VTA and SNc DA neurons would provide a second alternative. It is possible that there is local connectivity between midbrain DA cells and independently from inputs and outputs the activation of one population would induce the modulation of the other (Figure 3B), although local synaptic connectivity between DA neurons has not been experimentally demonstrated. Along this line of thought, the co-released glutamate could act as an activator of neighboring DA cells. Once a reward signal is processed at the level of the VTA, it would then automatically recruit the neighboring SNc motor component and vice versa. The close proximity of the two structures and technical limitations to study this hypothesis render this topic so far uncharted territory. Paired recordings *in vitro* could help answering this open question and provide the first evidence investigating DA-DA interactions at the level of the midbrain. A follow up step would need to characterize whether this DA-DA interaction is crucial for the reward-motor pairing during positive reinforcement learning.
3. A third possibility is that the synchronization of rewarding and motor components of reinforcement happens downstream of the SNc and VTA DA neurons (Figure 3C), at the level of their functional output targets. In addition to the expected motor effects, optogenetic self-stimulation of dMSNs in the DMS leads to reinforcement of actions while iMSNs self-stimulation triggers avoidance of actions (Kravitz et al., 2012). This highlights how direct activation of the postsynaptic targets of SNc is sufficient to produce both motor and reward/avoidance phenotypes, supporting the theory of local connectivity mechanisms that aid in the synchronization of the two behavioral sub-components. Furthermore, it was shown that the acquisition and expression of the reinforcement was insensitive to DA pharmacology (Kravitz et al., 2012). These experimental observations support a mechanism of action that recruits specific SNc and VTA sub-circuits downstream and independently from the release of DA.

### Dopamine-Acetylcholine Interaction

SNc and VTA DA are crucial in all motor and reinforcement-related behaviors. Without DA there is no learning. Once more this notion is supported by a series of experimental and clinical observations. In PD, cholinergic markers are significantly reduced. Interestingly, ACh and DA share several characteristics aside from suffering a decrease in PD. Both are critically involved in learning processes, cholinergic interneurons (CINs) of the striatum also undergo firing pattern changes during associative learning as do midbrain DA neurons. Both neuromodulatory systems interact at the level of the SNc, VTA and striatum, and both cell types are able to co-release main neurotransmitters.

In the clinical field, six different stages correlating the anatomical extent of the pathology with the development of the symptoms (Braak et al., 2004) are used to describe the progression of the disease. SNc DA degeneration has been reported to occur only during stage 3, together with the loss of cholinergic neurons in the nucleus basalis of Meynert (Nakano and Hirano, 1984; Braak et al., 2004) and in the PPN (Hirsch et al., 1987). In addition, there is evidence of a concurrent reduction in cortical and striatal cholinergic markers. Both the cell death and the reduction in cholinergic markers are strongly correlated with a decline in cognitive function assessed by memory tests in PD patients (Perry et al., 1985). To contrast the cognitive dysfunction in PD, pharmacologically addressing the loss of cholinergic neurons with a cholinesterase inhibitor such as rivastigmine has proven effective (Emre et al., 2004). Alternatively, counterbalancing the reduced DA influence on striatal cells, by pharmacologically blocking cholinergic receptors improves some motor symptoms such as tremor. Unfortunately, cognitive side effects have been described, particularly affecting learning and memory ability (Little et al., 1998) as well as cognitive flexibility (Van Spaendonck et al., 1993). Such contrast in the effects of ACh mediated therapies raises the possible need of a reinterpretation of clinical data and nicotinic vs. muscarinic receptors may end up being orthogonally affected by these therapies. DA replacement therapy with the DA precursor, L-DOPA, or with DA agonist has been shown to increase the risk of developing cognitive symptoms following a prolonged use (Alty et al., 2009; Brusa et al., 2013). This intricate interaction between both neuromodulatory systems has proven counterproductive for pharmacological therapies where often the amelioration of certain aspects of the disease brings undesired side effects. Better understanding this interaction at a molecular and circuit level will help design novel and more specific approaches that would avoid these undesired side effects.

Although there are other sources of ACh in the BG, we will focus on the two most prominent and better characterized to date, the striatum and the PPN.

Striatal ACh is supplied by an intrinsic neural network of large-sized CINs. They comprise only 1%–2% of the striatal cellular population (Kawaguchi, 1992, 1993). They display two types of rhythmical discharges, tonic and bursting firing. *In vivo* recording studies surprisingly revealed that CINs do not respond to movement *per se* (Crutcher and DeLong, 1984a,b). They exhibit a very special pattern of activity with a pause in firing that is acquired in response to sensory stimuli that predict motivationally significant outcomes (positive or negative). This response is lost when this association is extinguished (Kimura et al., 1984; Aosaki et al., 1994; Apicella et al., 1997; Apicella, 2002; Morris et al., 2004). The pause response is hence considered a neural correlate of classical conditioning. DA neurons show similar properties, they are first excited by a reward and if the reward is preceded by an associated salient stimulus they respond to the conditioned stimulus and not any more to the reward itself (Schultz et al., 1997). The pause in the CINs’ tonic firing is temporally correlated to the increase in firing of DA neurons. The response of both neurons encodes the probability of reward: the DA neurons respond to reward mismatch between expectation and outcome, while the cholinergic pause represents the expected timing of presentation reward (Fiorillo et al., 2003; Morris et al., 2004).

The cholinergic response is quite homogeneous; however, it can be preceded by brief increase in firing rate or followed by a rebound excitation. Several studies have provided evidence for intrinsic channel properties being responsible for this flanking excitatory activity (Wilson and Goldberg, 2006). Some studies, on the other hand, report the involvement of cortical (Reynolds and Wickens, 2004) or thalamic inputs in the generation of these phases (Matsumoto et al., 2001). So far, no consensus has been reached to determine whether this peculiar response is generated purely due to intrinsic properties, is network derived, or if both aspects are necessary, particularly for the learning ability the pause represents.

CIN’s bursting and pause responses were shown to be tightly synchronized population-wise (Raz et al., 1996) and such features are crucial for learning mechanisms, specifically for synaptic plasticity. In addition, ACh and DA interact closely as evidenced by a very well cahracterized anatomical and functional distribution. CINs express the D1 and D2-like DA receptors (Wieland et al., 2014), conversely DA nerve terminals express nACh receptors (Zoli et al., 2002; Figure 2). Photostimulation of DA neurons was shown to drive regionally heterogeneous synaptic responses in striatal CINs, including an acute excitation followed by an inhibition in the Nac medial shell (mediated by the co-released glutamate), a pause in the Nac core and the dorsal striatum (DS; both DA dependent; Chuhma et al., 2014). The pause response in the DS was also reported to be partly mediated by GABA (Straub et al., 2014). Conversely, the synchronized activation of CINs (electrical or optical stimulation) and hence an increased ACh level has been shown to induce the release of DA in the striatum as measured by fast-scan cyclic voltammetry (Surmeier and Graybiel, 2012; Threlfell et al., 2012). Such a synchronized CIN activation was also reported to mediate the co-release of GABA from the DA terminals to disynaptically inhibit MSNs (Nelson et al., 2014) highlighting how the dopaminergic and cholinergic systems can have a mutual modulation at the level of the striatum.

The acquisition of motor and cognitive action sequences depends on this intra-striatal DA-ACh balance (Calabresi et al., 2000). Through the interaction with DA, ACh has an integrative and modulatory role in the BG circuitry (Kaneko et al., 2000). During bursting, the increased level of ACh leads to M1 receptor activation which induces long term potentiation (LTP), on the opposite during the pause when ACh levels and M1 receptor activation decreases, long term depression (LTD) is favored (Centonze et al., 1999, 2003; Pisani et al., 2005; Bonsi et al., 2011). Furthermore, the reduced level of ACh leads to a diminished activation of nACh receptors on DA terminals, ultimately disinhibiting DA release (Rice and Cragg, 2004; Zhang and Sulzer, 2004; Cragg, 2006; Exley and Cragg, 2008). Hence, the concomitant drop of ACh and increase of DA facilitates D2 mediated LTD on MSNs. In this context, the pause response, which is concurrent with increased firing of SNc DA cells (Morris et al., 2004), may represent a period of enhanced LTD. Since the pause increases as the association learned by classical conditioning is strengthened (Aosaki et al., 1994), it has been proposed as a mechanism to signal that the association is well learnt, and so, the striatal network should refrain from engaging in any other form of LTP which normally encodes initial phase of learning.

Heterogeneity of CINs has not been reported to the extent that it has for DA. To the best of our knowledge the transcriptome and proteomic content of CINs have not been thoroughly investigated. At the functional level, although the pause pattern has been observed in response to both a reward and a punishment, single CINs seem to be able to encode both rewarding and aversive stimuli (Apicella, 2002).

Another source of ACh, able to modulate BG function has been described, the PPN. This brain region holds cholinergic projecting neurons that in contrast to the striatal CINs, co-release glutamate or GABA. Furthermore, cholinergic PPN neurons modulate DA cells and hence represent another point of DA-ACh interaction. SNc DA cells represent an important output target of PPN ACh neurons (Woolf and Butcher, 1986; Beninato and Spencer, 1987, 1988). A certain degree of heterogeneity has been reported; specifically, the projection onto the lateral SNc DA neurons is mainly excitatory (nicotinic or glutamatergic) and promotes locomotion whereas the medial SNc DA neurons receive mostly cholinergic mediated GABAergic transmission, which inhibits motor activity (Estakhr et al., 2017).

The PPN was also reported as a monosynaptic input of VTA DA neurons in mapping studies (Henderson and Sherriff, 1991; Watabe-Uchida et al., 2012), in fact, ascending ACh PPN projections target DA neurons in the SNc and VTA following a topographical gradient (Mena-Segovia et al., 2008). Optogenetic release of ACh from the PPN was shown to increase the bursting activity of VTA DA neurons. This connection is excitatory and sufficient to increase motor activity (Dautan et al., 2016). The potential involvement of ACh PPN modulation of SNc and VTA DA neurons in reinforcing behavior remains unclear.

ACh PPN neurons also project to the striatum and the synapses formed by the local vs. projecting ACh neurons can be morphologically differentiated (Dautan et al., 2014). The distinct activity of striatal CINs and brainstem ACh projecting-cells during reward-related paradigms may suggest that the two systems play different but complementary roles in the processing of information in the striatum. Towards this line of thought, it was suggested that the release of ACh from CINs is tonic and interrupted by behaviorally relevant events, whereas the release of ACh from the PPN would be phasically increased during salient events (Dautan et al., 2014). The functional relevance of the two distinct sources of ACh in the striatum remains unresolved experimentally. The impact of the co-release neurotransmitters glutamate and GABA is also not clearly evaluated, and so far, PPN but not striatal CINs have been shown to degenerate in PD, further highlighting the differential role of these two sources of ACh, and the importance of thoroughly characterizing them in an attempt to find PD predictive markers or features that could be exploited in therapeutic approaches.

The progression of PD related lesions, which was reported to start in the lower brainstem and olfactory bulb, subsequently moving up to the upper brainstem first invading the PPN followed by the midbrain (Braak et al., 2004), represents an important cue to follow. It is interesting to correlate such sequence of degeneration with the circuit-connectivity knowledge recently acquired, specifically PPN neurons projecting to and modulating the midbrain. In other words, even though the cause of the degeneration is still unknown, it is tempting to hypothesize that the spreading of the disease follows functional synaptic networks through mechanisms that still need to be evaluated but might be similar to those described in protein misfolding diseases (Pecho-Vrieseling et al., 2014).

## Conclusion

Neuromodulation of the BG represents a critical aspect in the generation of its different functions. Midbrain DA neurons show a high level of heterogeneity when considering biochemical markers, electrophysiological properties, co-release of main neurotransmitters, input-output connectivity and its related specialized behavioral functions. Such aspects driving a functional heterogeneity, although not exhaustive, raise the question of how the neurobiological substrates of distinct behavioral sub-domains cooperate to generate complex responses to environmental stimuli and ultimately maintain homeostasis. Hence, acquiring more knowledge about the neuromodulation of BG from the circuit/function point of view is critical to better understand how BG functions are generated in physiological conditions. For example, *in vivo* calcium imaging of DA and ACh terminals arising from functionally defined cell populations, in different projection sites, within varied behavioral contexts would provide better resolved information about the contribution of these two neuromodulators to naturalistic actions. In a similar way, site and function specific targeting of axons in a loss of function experimental design would complement the previous information and provide causal evidence of BG control of actions. Once this information is available, it would be possible to design medical approaches that can treat highly specific aspects of neurodegenerative disorders leaving all other functions intact. For example, different strategies could be taken to treat the motor symptoms of PD without carrying a cognitive decline and vice versa.

In the last 15 years, the development of circuit dissection tools and techniques have provided an incredible amount of knowledge at a systems neuroscience level. Unfortunately, very little of this information is reflected or used at the translational or clinical domains. It will be a long road to accommodate these new findings to refine the current pharmacological and electrical treatments, but the modification of sub-circuits in PD is an indispensable aspect that must be considered in the design of optimally targeted therapies. A significant improvement in therapeutical strategies may reside in the development of circuit and cell type specific pharmacological compounds. As of today, some pioneering studies are trying to address this challenge combining site specific deep brain stimulation and pharmacology (Creed et al., 2015).

## Author Contributions

Each author contributed equally to the design and writing of the review.

## Conflict of Interest Statement

The authors declare that the research was conducted in the absence of any commercial or financial relationships that could be construed as a potential conflict of interest.
